# When do patients resume driving after fast-track hip and knee arthroplasty: a cohort study of 408 patients

**DOI:** 10.2340/17453674.2026.46649

**Published:** 2026-09-24

**Authors:** Oddrún DANIELSEN, Jens LAURITSEN, Martin LINDBERG-LARSEN

**Affiliations:** Department of Orthopaedic Surgery and Traumatology, Odense University Hospital and Orthopedic Research Unit, Department of Clinical Medicine, University of Southern Denmark, Odense, Denmark

## Abstract

**Background and purpose:**

Only scattered knowledge is available on when patients resume driving following surgery. We primarily aimed to investigate the timing of driving resumption and secondarily to investigate the influence of patients’ and surgical factors after fast-track hip and knee arthroplasty.

**Methods:**

This prospective cohort study included patients undergoing primary total hip arthroplasty (THA), total knee arthroplasty (TKA), and medial unicompartmental knee arthroplasty (mUKA) in a fast-track setup at Odense University Hospital, Denmark, from September 2022 to July 2023. Eligible patients received a survey 3 to 12 months post-discharge, regarding timing of driving resumption.

**Results:**

408 patients received the survey with a response rate of 88%. Within 2 weeks postoperatively, 13% (95% confidence interval [CI] 8–20) of THA, 11% (CI 6–19) of TKA, and 28% (CI 18–40) of mUKA resumed driving, which increased to 77% (CI 69–84) of THA, 65% (CI 55–74) of TKA, and 80% (CI 68–88) of mUKA by week 6. Median time to driving resumption was 6 weeks for THA and TKA, and 4 weeks for mUKA. The overall median time to resume driving was shorter for patients who underwent surgery on the left leg (4 weeks) than those who underwent surgery on the right leg (6 weeks). Female patients had a higher odds ratio of delayed (> 6 weeks) driving resumption (OR 3, CI 2–5).

**Conclusion:**

Overall, 65–80% of patients resumed driving within 6 weeks with median time being shorter (4 weeks) among mUKA patients and patients who underwent surgery on the left leg. Female sex was associated with delayed resumption of car driving beyond 6 weeks postoperatively

Patients undergoing elective surgery often have expectations and a desire to swiftly resume normal activities, including driving. A rapid return to driving facilitates re-engagement with social networks, leisure activities, and work. Safely operating a vehicle depends on several factors, including the effects of anesthesia, opioid use, and immobilized joints [1]. Healthcare professionals are legally obligated in many countries to determine and report if a patient is unfit to drive, but there is little scientific evidence on individual-level assessment [2].

Previous studies on this subject are limited and heterogeneous, with most studies based on car simulator data to investigate changes in brake reaction time [1,3-6], a critical factor for driving safety. However, the results have shown considerable variability in how quickly brake reaction time normalizes postoperatively. Over the past decade, advancements in the implementation of fast-track protocols [7,8] have accelerated the recovery times following hip and knee arthroplasty, potentially also impacting driving resumption postoperatively. This raises the question of patients’ actual behavior regarding driving resumption today.

Hence, the primary aim of our study was to investigate the timing of resumption of driving, and the secondary aim to report the role of patient and surgical characteristics following fast-track hip and knee arthroplasty. An additional outcome of interest was the proportion of postoperative self-reported traffic incidents as a car driver.  

## Methods

### Study design

This study was based on a cohort of prospectively included consecutive hip and knee arthroplasty patients receiving a postoperative questionnaire on car driving resumption after surgery. The reporting of this study adheres to The REporting of studies Conducted using Observational Routinely-collected Data (RECORD) guideline [9].

### Setting

This was a single-center study on patients undergoing primary hip and knee arthroplasty at Odense University Hospital, Svendborg, Denmark. The patients were operated on in a fast-track set-up with early mobilization on the day of surgery and discharge on the day of surgery in at least 30% of cases [8]. Odense University Hospital, Svendborg, is a part of the multicenter collaboration “The Center for Fast-track Hip and Knee Replacement” following the same protocol for fast-track surgery [10,11]. The study period was from September 1, 2022, to July 31, 2023.

Prior to surgery, patients are informed that they may resume driving postoperatively after hip and knee arthroplasty once they can operate a vehicle safely without functional impairment of the operated-on limb and when they are no longer under the influence of opioids.

### Population

All included patients were treated with primary, unilateral total hip (THA), total knee (TKA), or medial unicompartmental knee arthroplasty (mUKA). The patients were screened for day-case eligibility using well-defined inclusion and exclusion criteria and discharged when fulfilling predetermined discharge criteria, resulting in a mean hospital stay of 0.7 days postoperatively. The questionnaire was conducted and distributed through REDCap, and was sent to the patients’ E-boks, an authorized digital mailbox. Patients without an authorized digital mailbox were therefore excluded.

The patients included in this study cohort were also reported on in previous publications, but in another context and with a different endpoint [8,12].

### Surgical procedures

Patients were operated on according to a standard protocol with posterior approach for THA and medial parapatellar approach for both TKA and mUKA (shorter incision). All surgical procedures were performed or supervised by experienced specialized arthroplasty surgeons (3 hip surgeons and 3 knee surgeons) performing more than 150 procedures annually. Local anesthesia with a minimum 150 mL ropivacaine 2 mg/mL was used in knee arthroplasties but not in hip arthroplasties.

### Data sources

Trained research staff prospectively collected data, with physician support available when required. Data collection included both patient-reported information on patient demographics, and detailed clinical data extracted from electronic patient records regarding patient demographics, co-morbidities, medication, and surgical characteristics [11]. All data was securely stored in an online REDCap database (https://project-redcap.org/) provided by the Open Patient Data Explorative Network (OPEN) at Odense University Hospital [13]. A questionnaire was conducted and distributed through REDCap to the patients’ E-boks, an authorized digital mailbox, on October 23, 2023. The questionnaire targeted all patients who had undergone primary THA, TKA, and mUKA within 3 to 12 months prior to receiving questionnaires on car driving resumption, ensuring a minimum postoperative recovery period of at least 3 months. The questionnaire consisted of 3 questions: (i) possession of a driving license and preoperative driving habits, (ii) timing of driving resumption postoperatively, and (iii) involvement in any postoperative traffic incident as the driver of a car (Table S1, see Supplementary data). All patients were assured that their responses would be anonymized and used exclusively for research purposes, with no risk of legal consequences.

### Variables

Demographic variables were age (continuous variable), sex (M/F), body mass index (BMI), cohabitation (cohabiting, living alone), Clinical Frailty Scale (1–8) [14], preoperative psychiatric medication (yes/no), and preoperative opioid use (yes/no).

Psychiatric medications were defined according to ATC codes N0^5^ and N0^6^, including antipsychotics, antidepressants, anxiolytics, and mood stabilizers.

### Outcomes

The primary outcome variable was the time (in weeks) to resumed car driving following THA, TKA, and mUKA. The primary outcome was analyzed overall and for each side individually. The secondary outcome of interest was risk factors associated with delayed resumption of car driving of more than 6 weeks postoperatively. An additional outcome of interest was the proportion of postoperative self-reported traffic incidents as a car driver.

### Statistics

Descriptive statistics were provided with means and standard deviations (SD) for continuous variables that followed a normal distribution. For non-normally distributed continuous variables, medians along with interquartile ranges (IQR) were presented. Categorical were described as proportions with 95% confidence intervals (CI) and analyzed using the Clopper–Pearson method. A multivariable logistic regression analysis was conducted to assess the association between various risk factors and the delay in resuming car driving > 6 weeks postoperatively, expressed as odds ratios (OR) with a CI and a P value. Variables included in the regression analysis were selected a priori based on clinical plausibility and previous literature, supplemented by our hypotheses regarding factors potentially influencing delayed resumption of driving. Assumptions for the logistic regression model were checked with the Hosmer–Lemeshow goodness-of-fit test and found to be acceptable (P = 0.4). All statistical tests were two-sided, with a significance level of 0.05. The cumulative incidence for driving resumption was calculated using a simple cumulative proportion approach. Cumulative incidence curves were constructed by calculating the cumulative proportion of patients who had resumed driving by each postoperative week. Exact 95% confidence intervals were calculated for each weekly cumulative proportion. Missing data was low (< 5% for all variables) and was assumed to be missing completely at random or missing at random. Descriptive analyses were conducted using available-case analysis, whereas regression analyses were based on complete-case analysis. Data was analyzed using Stata Statistical Software: Release 18 (StataCorp LLC, College Station, TX, USA).

### Ethics, registration, funding, and disclosures

Prior to surgery, all patients received both written and oral information on the project. Written informed consent was required to obtain full access to patient files and to send out questionnaires The fast-track set-up was implemented as standard of care outlined in the protocol [11]; as such no ethical approval was necessary for this study. The study was preregistered at ClinicalTrials.gov (NCT05613439) and in the Region of Southern Denmark, with approval granted for data management (Journal No 22/39454). The Center for Fast-Track Hip and Knee Replacement received funding from the NOVO Nordisk foundation (Grant number: NNF21SA0073760) to facilitate the organizational setup. Salary for the PhD student (OD) was provided through funding from the Candys Foundation, University of Southern Denmark and Region of Southern Denmark. Complete disclosure of interest forms according to ICMJE are available on the article page, doi: 10.2340/17453674.2026.46649

## Results

**Figure 1:**
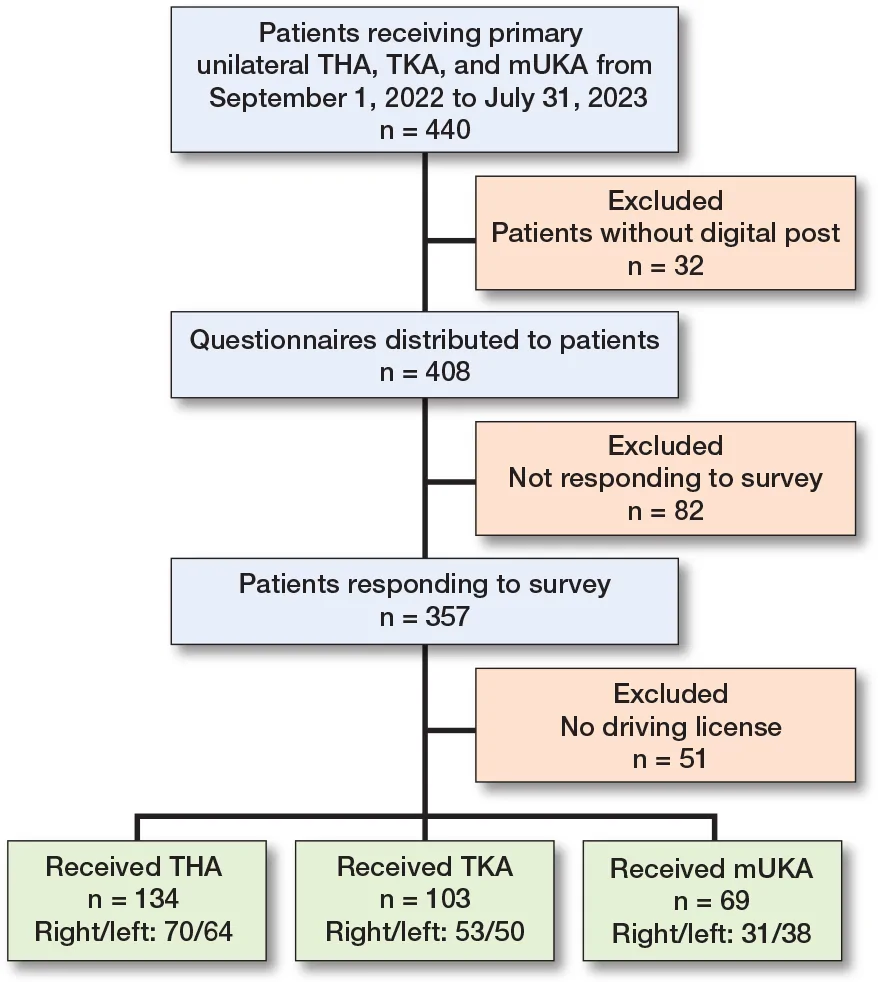
Flowchart of patient inclusion in the study. mUKA = medial unicompartmental knee arthroplasty; THA = total hip arthroplasty; TKA = total knee arthroplasty.

408 patients received the survey through their mailbox and 357 patients (88%) responded to the survey. 51 patients (14%) had no driver’s license and were excluded, hence patients with 134 THA, 103 TKA, and 69 mUKA were included (Figure 1). Baseline characteristics were comparable across all 3 arthroplasty groups (Table 1).

**Table 1:** Patient demographics. Values are count (%) or as specifed

Factor	Overall n = 306	THA n = 134	TKA n = 103	mUKA n = 69
Age, mean (SD)	70 (9.5)	70 (10)	70 (9.1)	71 (9.0)
Sex				
Female	165 (54)	74 (55)	58 (56)	33 (48)
Male	141 (46)	60 (45)	45 (44)	36 (52)
BMI, mean (SD)	29 (5.5)	28 (4.7)	31 (6.0)	29 (5.5)
Cohabitation				
Cohabiting	225 (74)	102 (76)	73 (71)	50 (73)
Living alone	75 (25)	27 (20)	29 (28)	19 (28)
Missing	6 (2.0)	5 (3.7)	1 (1.0)	–
CFS, mean (SD)	2.5 (0.7)	2.4 (0.6)	2.6 (0.8)	2.4 (0.6)
Preoperative				
psychiatric medication	38 (12)	10 (7.5)	17 (17)	11 (16)
opioid use	38 (12)	19 (14)	10 (9.7)	9 (13)
Surgical side				
Right	154 (50)	70 (52)	53 (51)	31 (45)
Left	152 (50)	64 (48)	50 (49)	38 (55)

BMI = body mass index, CFS = Clinical Frailty Scale [14]; mUKA = medial unicompartmental knee arthroplasty; THA = total hip arthroplasty; TKA = total knee arthroplasty.

### Driving resumption patterns following hip and knee arthroplasty

**Figure 2:**
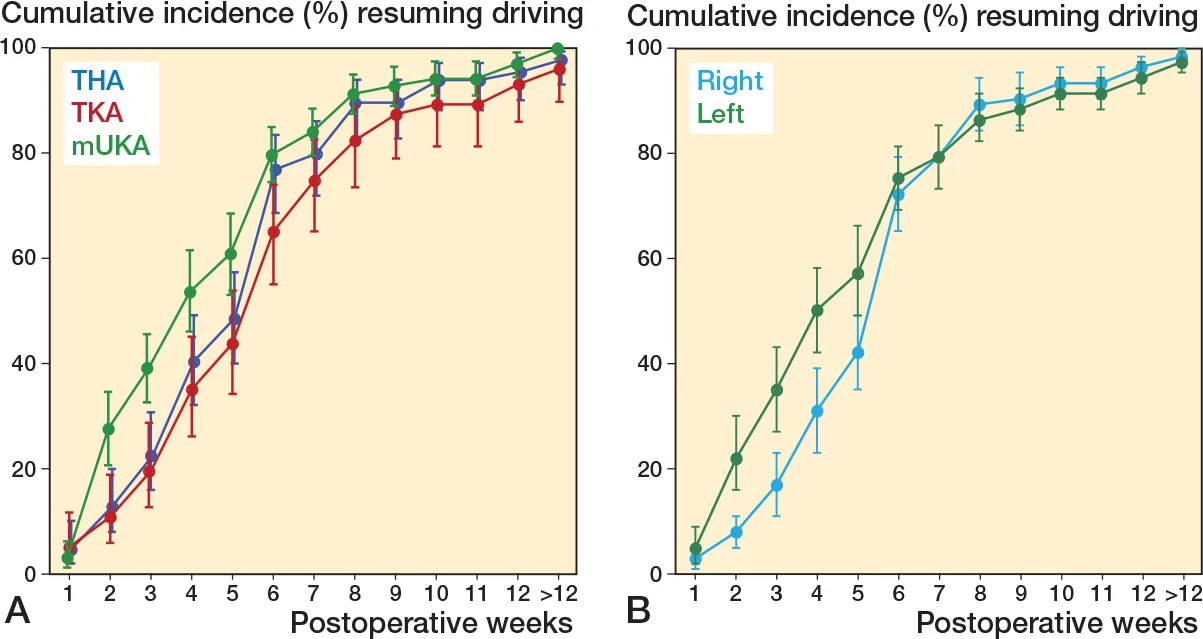
Cumulative incidence (%) of patients resuming driving each postoperative week depending on type of arthroplasty (A), i.e., total hip arthroplasty (THA), total knee arthroplasty (TKA), and medial unicompartmental knee arthroplasty (mUKA) and depending on surgical side (B). Whiskers are 95% confidence intervals. The median time for driving resumption after THA was 6 weeks, after TKA 6 weeks, after mUKA 4 weeks after surgery on right leg 6 weeks and after surgery on left leg 4 weeks.

After THA, 13% (CI 8–20) resumed driving within 2 weeks and 77% (CI 69–84) within 6 weeks. After TKA, 11% (CI 6–19) resumed driving within 2 weeks and 65% (CI 55–74) within 6 weeks. Following mUKA, 28% (CI 18–40) resumed driving within 2 weeks and 80% (CI 68–88) within 6 weeks. The median time to resuming driving was 6 weeks for both THA and TKA, and 4 weeks for mUKA (Figure 2A).

22% (CI 16–30) of patients who underwent surgery on the left leg resumed driving within 2 weeks whereas 8% (CI 5–14) of patients who underwent surgery on the right leg resumed driving in the same period. From week 6 onwards the proportion of driving resumption was approximately similar for patients who underwent surgery on the left and right leg. The median time to driving resumption was shorter among patients who underwent surgery on the left leg (4 weeks, CI 3–5) compared with those who underwent surgery on the right leg (6 weeks, CI 5–7) (Figure 2B).

### Factors influencing delay in resuming car driving

Female patients had a higher odds ratio of delayed car driving resumption than male patients (OR 3, CI 2–5). No other examined factor demonstrated a statistically significant association with delayed resumption of driving (Table 2).

**Table 2:** Determinants of delay in resuming car driving 6 weeks post-surgery

Risk factor	Odds ratio (CI)	P value
Age (ref. < 75 years)		
> 75 years	1.0 (0.6–1.7)	0.9
Sex (ref. male)		
Female	2.8 (1.6–4.8)	< 0.001
Type of surgery (ref. mUKA)		
THA	0.9 (0.5–1.8)	0.8
TKA	1.7 (0.9–3.3)	0.1
Surgical side (ref. left)		
Right	1.1 (0.7–1.8)	0.7
CFS (ref. 1–3)		
≥ 4	1.6 (0.6–4.1)	0.3
Psychiatric medication (ref. no)		
Yes	1.5 (0.7–3.3)	0.3
Preoperative opioid use (ref. no)		
Yes	0.5 (0.2–1.1)	0.1

For abbreviations, see Table 1.

### Postoperative automobile traffic incidents

Only 2 individuals (0.7%, CI 0.1–3) responded “yes” to having been involved in a car traffic incident postoperatively.  

## Discussion

To our knowledge, this is the first study to report the actual timing of driving resumption following hip and knee arthroplasty within a fast-track setup involving short hospital stays of 0–1 days. In this prospective cohort study investigating the timing of driving resumption after arthroplasty, we found that more than twice as many mUKA patients resumed driving during the first 2 weeks compared with THA and TKA. The median time to resuming driving was 6 weeks for both THA and TKA, and 4 weeks for mUKA. The median time to resume driving was shorter among patients who underwent surgery on the left leg (4 weeks) compared with those who underwent surgery on the right leg (6 weeks) across all 3 surgical types. Female sex was associated with delayed car driving beyond 6 weeks postoperatively.

Notable advancements have been made in the field of arthroplasty over the past decade, including implementation of fast-track protocols [7,8] resulting in early mobilization (intended < 4 h after surgery) and faster recovery times.

Previous studies on driving resumption after hip and knee arthroplasty have reported variability in the timing [1,3-6,15,16]. We found the median time to driving resumption after THA to be 6 weeks and 77% of patients were driving within 6 weeks. A meta-analysis of 8 studies on patient-reported return to driving after THA similarly found that most patients resumed driving within 6 weeks postoperatively. However, the quality of evidence was graded as “very low” or “low” across all included studies, primarily due to publication bias [3]. Our findings for driving resumption after TKA were comparable to those for THA, with a median time of 6 weeks and 65% of patients resuming driving within this timeframe. Similarly, Ellanti et al. reported that most patients resumed driving within 6 weeks after a TKA in a patient-reported study. However, this study excluded patients who had not resumed driving within 6 months postoperatively [15].

We found that patients receiving mUKA were associated with earlier driving resumption than patients receiving THA and TKA. The mUKA procedure is generally considered a less invasive procedure, and less pain and faster mobilization could be expected. However, the observed differences may also reflect underlying differences between patient groups not fully captured in this present study. To our knowledge, the timing of actual driving resumption after mUKA has not previously been reported. Liebensteiner et al. recommend refraining from driving after UKA during the first 6 postoperative weeks due to impaired brake response [6]. However, their study is a simulation study with a small sample size, which complicates direct comparisons with our findings. In our study, the majority of mUKA patients resumed driving within 6 weeks (80%), and having no postoperative car traffic incidents, suggesting that it may be safe for these patients to resume driving shortly after the surgery.

Patients who underwent left-sided surgery resumed driving faster than those who had right-sided surgery, a finding consistent with prior studies [1,3-5,17]. One possible explanation for this difference is that many patients may have access to cars with automatic transmission gearboxes, which do not require the use of the left leg for operating the clutch pedal, thereby minimizing the impact of surgery on driving ability. In contrast, right-sided surgery directly affects the leg used for accelerating and braking in both automatic and manual cars, requiring a higher degree of mobility and control for safe vehicle operation. This distinction could likely account for the observed delay in driving resumption among patients with right-sided surgeries.

Female sex was associated with delayed resumption of driving in accordance with prior research [16].

The observed disparity between sexes may be explained by the fact that females tend to adopt a more cautious driving approach, engaging in less risky behavior, which could contribute to the delay in the resumption of driving [18,19]. Additionally, men may often display higher self-confidence in their driving skills, even when skill ratings are comparable [20]. This divergence in confidence levels might play a role in influencing the duration it takes for females to regain confidence and resume driving following joint replacement surgery [21].

### Strengths

The strengths of our study primarily lie in its prospective design with a well-defined setup. Additionally, the high response rate (88%) enhances the reliability and generalizability of the findings.

### Limitations

Recall bias may potentially affect the accuracy of the reported timing of resuming driving.

Our questionnaire did not include information on preoperative driving habits or driving experience, which may have influenced individual decisions regarding when to resume driving following surgery.

There are also certain analytical limitations to consider when interpreting the analysis of patient- and surgery-related factors in relation to driving resumption. Small subgroup sizes and the inclusion of multiple variables increase the level of uncertainty and the risk of false-positive findings.

Although variables such as preoperative psychiatric medication and preoperative opioid use were included to account for factors potentially influencing recovery and behavioral readiness, residual confounding cannot be excluded, as these variables may not fully capture underlying differences between patients. In addition, some degree of selection bias cannot be excluded, as patients were not randomly assigned to procedures, and differences in health status, activity level, and postoperative recovery may not be fully captured. Although no formal recommendations were given to patients regarding timing of driving resumption, the study may be biased by the implicit influence of healthcare providers’ standard practice and existing knowledge, which often suggests a 6-week postoperative recovery period. This factor may contribute to the observed trend where a predominant number of patients resumed driving at 6 weeks.

### Conclusion

Overall, 65–80% of patients resumed driving within 6 weeks. Median time for driving resumption was shorter among mUKA patients (4 weeks), and more than twice as many mUKA patients resumed driving during the first 2 weeks compared with TKA and THA patients. Median time to resume driving was also shorter among patients who underwent surgery on the left leg (4 weeks), and female sex was associated with delayed driving resumption beyond 6 weeks postoperatively.

*In perspective*, our findings demonstrate that it may be feasible and safe to resume driving within a few weeks following hip and knee arthroplasty. However, driving is a complex skill, and patient recommendations should be individualized based on a comprehensive assessment.

### Supplementary data

Supplementary Table S1 is available as supplementary data on the article home page, doi: 10.2340/17453674.2026.46649

OD undertook data gathering and processing. OD and MLL drafted the manuscript. All authors critically reviewed the manuscript and accepted the final version.  

## Supplementary Material



## Notes

Handling co-editors: Li Felländer-Tsai and Philippe Wagner

Acta thanks Sead Crnalic and Alexander Oxblom for help with peer review of this manuscript.

## References

[1] Van der Velden C A, Tolk J J, Janssen R P A, Reijman M. When is it safe to resume driving after total hip and total knee arthroplasty? A meta-analysis of literature on post-operative brake reaction times. Bone Joint J 2017; 99-B(5): 566-76. doi: 10.1302/0301-620X.99B5. 28455464

[2] European Commission: Directorate-General for Mobility and Transport, TRL, Monash University, BASt, Loughborough University, SWOV, Fernández-Medina K, Fildes B, Helman S, Oxley J, Vlakveld W, Weekley J. Study on driver training, testing and medical fitness – Final report. Luxembourg: Publications Office; 2017. Available from: https://data.europa.eu/doi/10.2832/446103

[3] Patel P V, Giannoudis V, Palma S, Guy S P, Palan J, Pandit H, et al. Doctor when can I drive? A systematic review and meta-analysis of return to driving after total hip arthroplasty. Hip Int 2023; 33(1): 17-27. doi: 10.1177/11207000219980. PMC9827492 33736494

[4] Muh S J, Shishani Y, Streit J, Lucas C, Sahgal V, Kraay M, et al. The impact of joint replacement on driver function and safety. Open J Orthop 2012; 2(3): 121-5. doi: 10.4236/ojo.2012.23022.

[5] Kirschbaum S, Fuchs M, Otto M, Gwinner C, Perka C, Sentürk U, et al. Reaction time and brake pedal force after total knee replacement: timeframe for return to car driving. Knee Surg Sports Traumatol Arthrosc 2021; 29(10): 3213-20. doi: 10.1007/s00167-020-06105-2. PMC8458211 32583024

[6] Liebensteiner M C Rochau H, Renz P, Smekal V, Rosenberger R, Birkfellner F, et al. Brake response time returns to the pre-surgical level 6 weeks after unicompartmental knee arthroplasty. Knee Surg Sports Traumatol Arthrosc 2014; 22(8): 26-31. doi: 10.1007/s00167-014-3050-1. 24832693

[7] Götz J, Maderbacher G, Leiss F, Zeman F, Meyer M, Reinhard J, et al. Better early outcome with enhanced recovery total hip arthroplasty (ERAS-THA) versus conventional setup in randomized clinical trial (RCT). Arch Orthop Trauma Surg 2024; 144(1): 439-50. doi:10.1007/s00402-023-05002-w. PMC10774173 37552325

[8] Danielsen O, Varnum C, Jensen C B, Jakobsen T, Andersen M R, Bieder M J, et al. Implementation of outpatient hip and knee arthroplasty in a multicenter public healthcare setting. Acta Orthop 2024; 7(96): 219-24. doi: 10.2340/17453674.2024.40185. PMC11077343 38715473

[9] Benchimol E I, Smeeth L, Guttmann A, Harron K, Moher D, Petersen I, et al. The REporting of studies Conducted using Observational Routinely-collected health Data (RECORD) statement. PLoS Med 2015; 6; 12(10): e1001885. doi: 10.1371/journal.pmed.1001885. PMC4595218 26440803

[10] Center for Fast Track – Hip and Knee Replacement. Available from: www.fast-track.health.

[11] Lindberg-Larsen M, Varnum C, Jakobsen T, Andersen M R, Sperling K, Overgaard S, et al. Study protocol for discharge on day of surgery after hip and knee arthroplasty from the Center for Fast-track Hip and Knee Replacement. Acta Orthop 2023; 20(94): 121-7. doi: 10.2340/17453674.2023.11636. PMC10028556 36942664

[12] Danielsen O, Jørgensen C C, Varnum C, Jakobsen T, Andersen M R, Bieder M J, et al. Day-case success or why still in hospital after total hip, total knee, and medial unicompartmental knee arthroplasties? Bone Jt Open 2024; 5(11): 977-83. doi: 10.1302/2633-1462.511. PMC11534455 39496281

[13] REDCap. Available from: https://open.rsyd.dk/opens-faciliteter/redcap

[14] Nissen S K, Fournaise A, Lauridsen J T, Ryg J, Nickel C H, Gudex C, et al. Cross-sectoral 307 inter-rater reliability of the clinical frailty scale: a Danish translation and validation study. BMC Geriatr 2020; 20(1): 443. doi: 10.1186/s12877-020-01850-y. PMC7640648 33143651

[15] Ellanti P, Raval P, Harrington P. Return to driving after total knee arthroplasty. Acta Orthop Traumatol Turc 2015; 49(6): 593-6. doi: 10.3944/AOTT.2015.14.0307. 26511684

[16] Lazar T A, Edelmann M, Awiszus F, Lohmann C. Surgical site, gender, and place of residence influence the time to resume driving after total joint arthroplasty. Arch Physiother 2021; 11(1): 16. doi: 10.1186/s40945-021-00111-4. PMC8240401 34183073

[17] Marques C J, Barreiros J, Cabri J, Carita A I, Friesecke C, Loehr J F. Does the brake response time of the right leg change after left total knee arthroplasty? A prospective study. Knee 2008; 15(4): 295-8. doi: 10.1016/j.knee.2008.02.008. 18407504

[18] Rhodes N, Pivik K. Age and gender differences in risky driving: the roles of positive affect and risk perception. Accid Anal Prev 2011; 43(3): 923-31. doi: 10.1016/j.aap.2010.11.015. 21376884

[19] Pan C, Ma J, Li Y, Lu Y, Shan L, Chang R. Sex difference in driving speed management: the mediation effect of impulse control. PLoS One 2023; 18(7): e0288653. doi: 10.1371/journal.pone.0288653. PMC10351722 37459346

[20] Wayne N L, Miller G A. Impact of gender, organized athletics, and video gaming on driving skills in novice drivers. PLoS One 2018; (13): (1): e0190885. doi: 10.1371/journal.pone.0190885. PMC5783381 29364957

[21] Maxwell H, Weaver B, Gagnon S, Marshall S, Bédard M. The validity of three new driving simulator scenarios: detecting differences in driving performance by difficulty and driver gender and age. Hum Factors 2021; 63(8): 1449-64. doi: 10.1177/0018720820937520. 32644820

