# Supplementary material for: When do patients resume driving after fast-track hip and knee arthroplasty: a cohort study of 408 patients
Source: Acta Orthop. 2026 Sep 24;97:715–20. doi: 10.2340/17453674.2026.46649 (PMC13602197; doi:10.2340/17453674.2026.46649)
Supplement: Supplementary file 1 [file ActaO-97-46649-s1.pdf]

Supplementary Table 1: Survey questionnaire - driving resumption after surgery

Question number	Survey question
1	Do you have a driver's license, and did you drive regularly before your surgery with an artificial hip or knee?<br>Answer: Yes / No
2	When did you resume driving after the surgery? (Please specify as accurately as possible)<br>Answer:<br>1 week after surgery,<br>2 weeks after surgery,<br>3 weeks after surgery,<br>4 weeks after surgery,<br>5 weeks after surgery,<br>6 weeks after surgery,<br>7 weeks after surgery,<br>8 weeks after surgery,<br>9 weeks after surgery,<br>10 weeks after surgery,<br>11 weeks after surgery,<br>12 weeks after surgery,<br>later than 12 weeks after surgery,<br>I have not resumed driving
3	Have you been involved in a car accident where you were the driver after your surgery? [a]<br>Answer: Yes / No

[a] All patients were assured that their responses would be used exclusively for research purposes and would not be subject to legal proceedings, particularly concerning question 3.
